# An Auto-Calibrating Knee Flexion-Extension Axis Estimator Using Principal Component Analysis with Inertial Sensors

**DOI:** 10.3390/s18061882

**Published:** 2018-06-08

**Authors:** Timothy McGrath, Richard Fineman, Leia Stirling

**Affiliations:** 1 Department of Aeronautics and Astronautics, Massachusetts Institute of Technology, 77 Massachusetts Avenue, Cambridge, MA 02139, USA; leia@mit.edu; 2 Harvard-MIT Division of Health Sciences & Technology, Massachusetts Institute of Technology, 77 Massachusetts Avenue, Cambridge, MA 02139, USA; rfineman@mit.edu; 3 Institute for Medical Engineering Sciences, Massachusetts Institute of Technology, 77 Massachusetts Avenue, Cambridge, MA 02139, USA

**Keywords:** inertial sensor, IMU, gait, knee flexion, knee extension, principle component analysis

## Abstract

Inertial measurement units (IMUs) have been demonstrated to reliably measure human joint angles—an essential quantity in the study of biomechanics. However, most previous literature proposed IMU-based joint angle measurement systems that required manual alignment or prescribed calibration motions. This paper presents a simple, physically-intuitive method for IMU-based measurement of the knee flexion/extension angle in gait without requiring alignment or discrete calibration, based on computationally-efficient and easy-to-implement Principle Component Analysis (PCA). The method is compared against an optical motion capture knee flexion/extension angle modeled through OpenSim. The method is evaluated using both measured and simulated IMU data in an observational study (*n* = 15) with an absolute root-mean-square-error (RMSE) of 9.24${}^{\circ }$ and a zero-mean RMSE of 3.49${}^{\circ }$. Variation in error across subjects was found, made emergent by the larger subject population than previous literature considers. Finally, the paper presents an explanatory model of RMSE on IMU mounting location. The observational data suggest that RMSE of the method is a function of thigh IMU perturbation and axis estimation quality. However, the effect size for these parameters is small in comparison to potential gains from improved IMU orientation estimations. Results also highlight the need to set relevant datums from which to interpret joint angles for both truth references and estimated data.

## 1. Introduction

Accurate measurement of human joint angles is central to the study of human biomechanics. Better biomechanical models and measurement systems enable more robust tools for interacting with and understanding human kinematics. For example, in rehabilitation, understanding human motion informs the plan of care. For sports applications, understanding motion can lead to improved strategy development. To this end, an accurate measurement of human joint angles is desired. This measurement is complicated, as human motion is characteristically nonlinear, non-smooth, and uncorrelated in time [1]. Among measurement methods, optical motion capture represents the current gold standard [2], although other computer vision approaches [3] do exist. Optical motion capture is accurate in triangulating reflective marker position in space, but interpretation of these data as human joint angles requires an assumed human model. An often-used model is OpenSim [4]. From markers set on major anatomical landmarks of the body, a least-squares optimization to fit a model may be performed to estimate the joint angles of interest.

This approach is financially expensive due to the equipment required to collect the data, and is cumbersome to post-process. The subject must be fitted in non-reflective clothing with markers placed precisely at anatomical landmarks. Any misplacement would manifest itself as an error in the estimation of joint angles. The infrared cameras are expensive, must be calibrated and oriented properly to view a necessarily-large capture volume, and suffer from marker dropout when the markers are occluded by the subject or another object. This approach is a valuable research tool, but can be inappropriate for the clinician, sports performance expert, or engineer when measurements need to be made at low cost and in the environment of interest.

Small, wearable accelerometer, gyroscope, and magnetometer packages, in contrast, offer an inexpensive way to make robust measurements. Decades of research in state estimation and filtering have enabled the appropriate mathematical models and techniques to estimate orientation [5] in three-dimensional space. The modern inertial measurement unit (IMU) is a common tool of motion measurement in non-deforming bodies. Inertial sensors have become common as navigation components for ballistic missiles, rockets, and aircraft, and are widely used today [6]. IMUs have become smaller [7], although magnetometers still, by nature, can become inaccurate in the presence of a disturbing magnetic field [8]. Applications of IMUs to human motion have also considered navigation through the environment [9]. Luinge et al. [10] first applied IMUs to the measurement of human limb orientation, albeit with limitations of only estimating tilt and requiring sensor calibration to a known position every 30 s. For normal upright gait, Mayagoitia et al. [11] used accelerometers and gyroscopes, precisely aligned to the body segment, to measure knee angle to a root-mean-square-error (RMSE) accuracy of at most 2.73${}^{\circ }$ (5.2% of gait task range of motion). The optical motion capture truth datum was assumed to be a rigid body angle between markers.

The knee’s primary degree of freedom is flexion/extension with a range of motion (ROM) of approximately 142.5${}^{\circ }$[12]. Minor degrees of freedom (DOF) of the knee include varus-valgus with ±5${}^{\circ }$ ROM [13] and internal-external rotation with ROM of 10${}^{\circ }$ [14]. Whether all three knee angles are needed or only the major flexion/extension DOF is application-dependent. Some clinical applications, such as understanding knee stability [15], require estimation of the degrees of freedom with lower range of motion. Inertial methods exist to estimate 3D knee angles [16,17], but evaluation of these methods in the minor degrees of freedom is difficult due to high measurement error relative to the ROM of the minor DOFs. In Favre et al. [16], for example, the RMSE of estimation of the knee varus-valgus angle was 35% of the joint’s ROM. Markers on the thigh and shank are subject to millimeters of soft tissue motion [18], which may confound calculation of joint angles. These ”truth” measurement errors, combined with the low range of the knee’s minor degrees of freedom, lead to a low signal-to-noise ratio when estimating vargus/valgus and internal-external rotation. Thus, caution must be taken when estimating these lower-ROM DOF via either inertial or optical methods. Estimation of flexion/extension is inherently more robust due to a higher range of motion to measurement error ratio.

Inertial measurement techniques inherently require an understanding of the relationship between the coordinate systems of the IMUs and the coordinate system of the joint in question. This relationship is established through a process of calibration, which primarily serves to align or measure relative orientation between the local frames of the IMUs and the joint’s coordinate system (JCS) [19,20,21]—such that computed angles may be interpretable as the anatomical joint angle. One method of calibration is through precise manual alignment of the sensors on the leg segment. Favre et al. [16,22] aligns the IMUs such that one of the axes of each IMU is aligned with the knee’s hinge axis while also employing a high-pass filter on the gyroscope data to minimize drift of the angle estimate. This mounting assumption is also seen in other research [10,11].

Alternate methods seek to use a functional calibration procedure to estimate the relationship between the body-mounted IMUs and the anatomical axes of the joint. Zhu et al. [23] decouples the degrees of freedom of arm motion on a human subject in order to excite the degrees of freedom separately, which allows a novel Kalman filter to estimate limb segment orientation. Luinge et al. [24] prescribes a pre-defined arm rotation procedure to determine the definitions of local rotation axes in the arm. Cutti et al. [25] and Favre et al. [17,26] each prescribe a functional calibration procedure for determination of leg segment-to-sensor orientation. Vitali et al. [27] uses two functional alignment motions to set the anatomical coordinate system of the thigh and shank. Cooper et al. [28] employs similar kinematic constraints of the knee hinge axis as above, but requires the knee hinge axis be provided by optical motion capture. These biomechanically-inspired methods offer powerful estimation tools over human-aligned techniques, especially in the presence of noisy sensors.

As IMU estimation of the knee joint angle has become more accurate, attention has shifted towards methods which require fewer functional calibrations and assumptions of alignments. Seel et al. [29,30] presents a novel method for estimating the knee axis through a formulated optimization problem, leveraging a kinematic argument of hinge joint motion. From this axis, the angle can be computed. Müller [31] extends this concept to the elbow, arguing that the relative angular velocity between two IMUs can be decomposed about the two major degrees of freedom of the elbow, and an optimization method is formulated to calculate those axes. These methods allow for less precise placement of the IMU on the body segment.

The relative angular velocity vector, as Müller implies, is a noteworthy quantity because it represents all relative motion between two IMUs. In the case of the knee, we simplify the knee to one degree of freedom and examine a method for estimating the primary flexion/extension axis. The relative angular velocity between an IMU on the shank and IMU on the thigh should generally point parallel to the direction of the knee hinge axis. This hinge axis should be well approximated by the principal component of the set of aforementioned relative angular velocity vectors. Principal Component Analysis (PCA) [32] is a general statistical technique to simplify high-dimensional data to a descriptive lower-dimensional structure. In biomechanics, it has been used to study coordination between time-series data [33]. Landry et al. [34] used PCA to study similarities in gait waveforms of knee angle between patients with osteoarthritis. Dillman et al. [35] used PCA in a similar way to study patients with Parkinson’s disease. In the case of physical data, like angular velocities, PCA offers a computationally-efficient method to find a primary directionality of the data.

In this paper, we examine the decomposition of relative angular velocity between a shank and thigh IMU into its primary component: rotation about the knee flexion/extension axis. PCA is used as a robust method to estimate this knee axis. Once the knee axis is estimated, a leg segment coordinate system may be defined for both the thigh and shank. The Euler angles between these two leg segment coordinate frames are then interpreted as the anatomical knee joint angles. For estimation of the knee’s hinge axis, PCA is more computationally-efficient than previously-proposed nonlinear least-squares optimization methods and is robust to the dual-direction representation of an axis. The method requires no assumption of mounting orientation on the body of the IMUs and requires no calibration to estimate the knee flexion/extension axis, any motion of the knee will suffice. A subset of this analysis, IMU-estimated knee angle for subjects 1 and 2, was previously presented [36]. This paper extends the previous work through analysis of additional subjects, developing an explanatory model of the error, and comparison of the method with ideal simulated IMU data and the method of Seet el al. [30].

To evaluate the proposed method, the knee flexion/extension axis was estimated during a timed-up-and-go task, which is commonly associated with increased fall risk in older adults [37]. Truth data were estimated using OpenSim modeling of optical motion capture data. We hypothesize (1) that the estimated knee joint angle from the proposed IMU method will be of similar error performance to other IMU-based measurement systems. (2) Accuracy of the method may be influenced by placement of the IMUs on the leg and soft-tissue noise; these factors are formalized and investigated further.

## 2. Problem Formulation

The human knee joint is commonly simplified as a single degree-of-freedom (DOF) hinge joint. We refer to this major rotation of the knee as flexion/extension. Other rotations of the knee include internal-external rotation and varus-valgus. These rotations are generally considered small, enabling the simplification of the knee as a hinge joint.

An IMU can be placed both distal and proximal to the knee joint, such that all relative motions between the IMUs are assumed to be due to knee flexion/extension at the joint. Small errors due to other motion of the knee or skin tissue artifacts are not modeled in this work. No assumption of alignment of the IMUs are made; the IMUs are free to be placed in any orientation on the thigh and shank, simply provided that one is distal and one is proximal to the knee joint.

The coordinate frame of the IMU on the thigh will be referred to as frame A, and, likewise, the frame of the IMU on the shank will be referred to as frame B. The flexion/extension axis of the knee will be referred to as axis ${\vec{a}}^{}$. As such, the anatomical axis ${\vec{a}}^{}$ expressed in frame B will be noted as ${\vec{a}}^{B}$. It can likewise be expressed in frame A as ${\vec{a}}^{A}$.

The relative angular velocity between the two IMUs can be expressed in frame B as:

(1) $${\vec{\omega }}_{rel}^{B}=-{\vec{\omega }}_{thigh}^{B}+{\vec{\omega }}_{shank}^{B}=-D_{A}^{B}{\vec{\omega }}_{thigh}^{A}+{\vec{\omega }}_{shank}^{B}.$$

The rotation matrix $D_{A}^{B}$ can be computed for every sample measurement *k* via simple rotation mathematics from the filtered orientation estimates of the individual IMUs. Multiple state estimators exist for the IMU sensor fusion for orientation problem [5,38,39,40]. In this work, an unscented Kalman filter, as implemented by the manufacturer of the IMU, was used to estimate IMU orientation.

In the case of a simple 1DOF hinge system with IMUs on either side of the joint, and a low-error measurement system (e.g., low-error gyroscopes and low-error state estimates of the IMU orientations, $D_{A}^{\mathrm{Global}}$ and $D_{B}^{\mathrm{Global}}$), the relative angular velocity vector between two IMUs, ${\vec{\omega }}_{rel}^{B}$, will point in the direction of the hinge axis of rotation. In practice, this remains true, with some (ideally small) errors in the direction of ${\vec{\omega }}_{rel}^{B}$ due to imperfect IMU orientation estimations and small perturbations of the IMUs on the skin surface due to deformable skin tissue artifacts. We assume that the knee anatomical axis is time-invariant, being fixed in time in both IMU coordinate systems. This assumption is consistent with biomechanical modeling techniques such as OpenSim. This assumption can be violated by off-axis rotations. In practice, these off-axis rotations can be considered a combination of unmodeled knee rotations (varus-valgus and internal-external rotation) and errors, namely IMU perturbations due to skin tissue artifacts.

A robust and computationally-efficient method to produce a best single estimate of the axis of rotation, ${\vec{a}}^{B}$, is through PCA. Operationally, the principle component of a PCA on a set of vectors will find the axis that minimizes the sum of the square errors of the orthogonal distances of individual vector measurements to the estimated principle axis. Since the set of vectors ${\vec{\omega }}_{rel}^{B}$ will generally point in the direction of the knee hinge axis (with some noise), PCA may yield a robust estimate of the anatomical knee hinge axis ${\vec{a}}^{B}$.

Note that this method could also be expressed in terms of frame A. Equation (1) could be written in terms of frame A to yield ${\vec{\omega }}_{rel}^{A}$, and PCA performed on ${\vec{\omega }}_{rel}^{A}$ to yield an estimate of the knee hinge axis in terms of frame A, ${\vec{a}}^{A}$. This approach is equivalent—the axes are related by the relative orientation as ${\vec{a}}^{A}=D_{B}^{A}{\vec{a}}^{B}$. This paper will estimate the knee axis and angle in frame B.

A further advantage of PCA to solve this axis estimation problem is the robustness to sign ambiguity of relative angular velocity, which yields a sign ambiguity of the knee hinge axis. In flexion, the relative angular velocity ${\vec{\omega }}_{rel}^{B}$ will point in one direction, and, in extension, ${\vec{\omega }}_{rel}^{B}$ will point in the opposite direction. Optimization or axis search methods may be confounded by this axis sign ambiguity. Since PCA only seeks to minimize the orthogonal distances of the measurements ${\vec{\omega }}_{rel}^{B}$ to a line estimate, it does not suffer from sign ambiguity.

Once the knee hinge axis ${\vec{a}}^{}$ has been estimated via PCA, the knee’s anatomical angles must be computed. This work implements an angle calculation method functionally similar to Laidig et al. [41] and Seel et al. [30]. A local leg segment coordinate system is defined as $S_{1}$ and $S_{2}$ for the thigh and shank leg segments. These coordinate systems are the IMU frames A and B rotated such that the *z*-axis aligns with the estimated knee hinge axis ${\vec{a}}^{A}$ or ${\vec{a}}^{B}$. For the purposes of flexion/extension calculation only, this segment frame can be related to the local IMU frame as a function of only estimated knee hinge axis as:

(2) $$D_{S_{1}}^{A}=\left[\begin{array}{ccc} {\vec{a}}_{y}^{A^{2}}+{\vec{a}}_{z}^{A^{2}} & 0 & {\vec{a}}_{x}^{A}\\ -{\vec{a}}_{x}^{A}{\vec{a}}_{y}^{A} & {\vec{a}}_{z}^{A} & {\vec{a}}_{y}^{A}\\ -{\vec{a}}_{x}^{A}{\vec{a}}_{z}^{A} & -{\vec{a}}_{y}^{A} & {\vec{a}}_{z}^{A} \end{array}\right]\;\forall {\vec{a}}^{A}\neq \left[\begin{array}{ccc} 1 & 0 & 0 \end{array}\right]$$

for the thigh segment, and similarly for the shank segment. This relationship can be deduced by setting the *z*-axis to ${\vec{a}}^{}$, the initial *x*-axis to [1 0 0], and then constructing an orthonormal *y* and *x*-axis. The relative orientation between these new frames $S_{1}$ and $S_{2}$ can then be calculated as:

(3) $$D_{S_{1}}^{S_{2}}=D_{S_{1}}^{A}{\left(D_{G}^{A}\right)}^{-1}D_{G}^{B}{\left(D_{S_{2}}^{B}\right)}^{-1},$$

where G is the common global frame. Finally, the relative orientation $D_{S_{1}}^{S_{2}}$ is then decomposed into *z*–*x*–*y* Euler angles. The first Euler angle, about the *z*-axis, is the knee flexion/extension angle, consistent with International Society of Biomechanics (ISB) JCS recommendations [19].

## 3. Materials and Methods

### 3.1. Participants

Fifteen subjects (7 male, 8 female, age = 20.7 ± 1.79 years) participated in the study. The protocol was approved by the Committee on the Use of Humans as Experimental Subjects at MIT (Protocol 1702862119). Exclusion criteria included (1) atypical neurological, heart, lung, or blood conditions; (2) surgeries performed within the previous six months; and (3) physical limitations which would require an assistive device.

### 3.2. Study Protocol

Each participant in the study completed three motion tasks: a 10-meter-walking task (10MWT), a Standing Balance task (SBT), and a Timed-Up-And-Go test (TUGT). Only the data from the TUGT was considered for this analysis. The TUGT began with the subject sitting on a stool. Then, the subject walked 3 m to a marker, turning around it, walking back to and sitting on the stool. Each participant completed 15 TUGT trials. The first five were practice trials, meant to teach the task, and the results were not processed for this analysis. The data from the final 10 TUGT trials that each participant completed were used for analysis. Each of the 15 subjects completed these 10 full trials, for 150 trials total. Four of the recorded trials were excluded due to missing or incomplete IMU data. Three trials were excluded due to poor/diverging IMU orientation estimates from the manufacturer’s onboard filter. This left 143 trials for analysis. The existence of poor IMU orientation estimates motivated the additional creation of a “simulated” set of IMU data from marker triads that had been placed on the IMUs.

**Figure 1:**
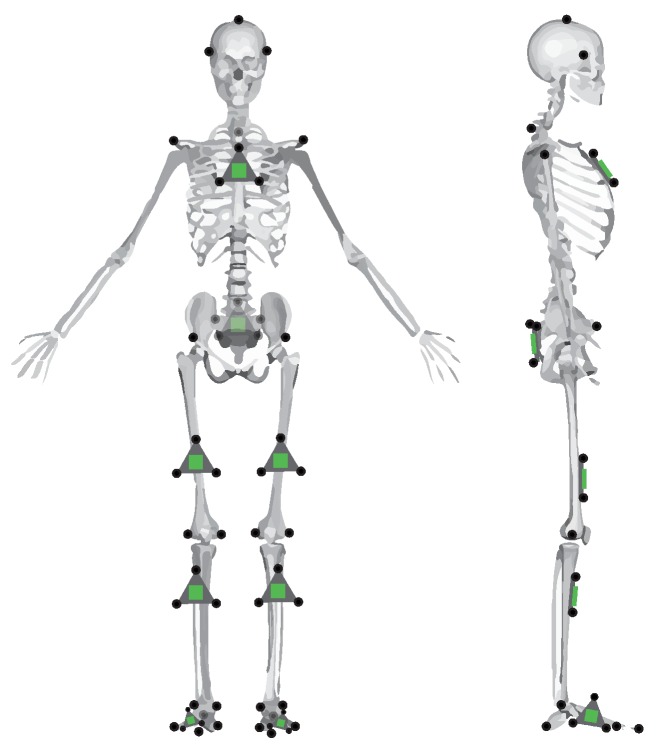
Placement of the reflective markers (black circles) and IMUs (green) on the subject. IMUs on the thigh and shank were not placed precisely, and location varied in the transverse plane.

Each subject was outfitted with a set of reflective motion capture markers and strap-on IMUs (Opal IMU, APDM, Inc., Portland, OR, USA). The position of these reflective markers and IMUs can be seen in Figure 1. As can be seen in the figure, some markers are placed on anatomical landmarks, independent of IMU positioning. These primary markers are placed according to a modified Cleveland Clinic lower body marker set for use in OpenSim inverse kinematic modeling. The position of the reflective markers was captured using a 14-camera Vicon motion capture system (Vicon Motion Systems, Inc., Los Angeles, CA, USA) at a sampling rate of 100 Hz.

### 3.3. Data Processing

The IMU contains dual 3-axis accelerometers (±16 g, ±200 g), a 3-axis gyroscope (±2000 deg/s), and a 3-axis magnetometer (±8 Gauss). The raw data from those sensors were fused for orientation using an unscented Kalman filter, as implemented by the manufacturer. The orientations from the two respective IMUs to a common global frame were used with the PCA method as described in Section 2 to estimate the knee hinge axis ${\vec{a}}^{}$ in each IMU frame. Then, the segment frames $S_{1}$ and $S_{2}$ were constructed from Equation (2). Finally, the relative segment frame orientation $D_{S_{1}}^{S_{2}}$ is computed from Equation (3), which encodes the estimated knee flexion/extension angle.

The optical motion capture marker data were low-pass filtered with a 30 Hz, 6th order Butterworth filter and then processed in OpenSim, with inverse kinematics computed via OpenSim’s gait 2392 model [42]. This solver minimizes error between the assumed placement of markers relative to an ideal biomechanical model (scaled for each subject) and the measured marker position from optical motion capture. The OpenSim subject model was acquired by scaling the generic OpenSim model for each subject, according to anthropometric measurements derived from the subjects’ marker data while static.

The IMU data were collected at 128 Hz, while the Vicon data were collected at 100 Hz. For discrete comparison of the datasets, the IMU-based knee angle estimates were downsampled to 100 Hz. The RMSE of the knee angle was calculated between the OpenSim-estimated truth data and the proposed method as:

(4a) $$\delta =\theta_{OpenSim,k}-\theta_{estimated,k},$$

(4b) $$RMSE_{absolute}=\sqrt{\frac{1}{N}\sum_{k=1}^{N}\delta^{2}},$$

(4c) $$RMSE_{zero-mean}=\sqrt{\frac{1}{N}\sum_{k=1}^{N}{\left(\delta -mean\left(\delta \right)\right)}^{2}},$$

where *N* is the number of data points included in the calculation. The absolute RMSE, Equation (4b), is the traditional calculation of RMSE. Any static offsets between the data being compared would manifest itself in absolute RMSE. However, it was found in the present study that there were static offsets between the OpenSim knee angle and the estimated knee angle from the IMUs. There are two primary sources of this static offset error: (1) human error in marker placement on anatomical landmarks and (2) the thigh and shank IMUs not lying in a plane perfectly parallel to the underlying OpenSim model bone segments. For the former, error in placement of the anterior/posterior superior iliac spine (ASIS/PSIS) markers, knee markers, and ankle markers would set a different angle datum when the rigid body angle between the markers is calculated and could influence the optimized OpenSim inverse kinematics solution. The latter source of error is due to the IMU not lying “flat” on the leg segment. If the thigh IMU is canted, for example, due to the shape of the quadriceps, the rigid body angle datum between the IMUs would be affected and would create a static offset from the assumed biomechanics model. In order to better understand the error of the proposed method without being confounded by the two aforementioned sources of static offset error, we also present zero-mean RMSE in Equation (4c). This equation is similar to normal RMSE, but the static offset between signals has been removed by subtracting the mean of the residuals from the residual data. This formulation permits a better comparison between the underlying waveforms.

**Figure 2:**
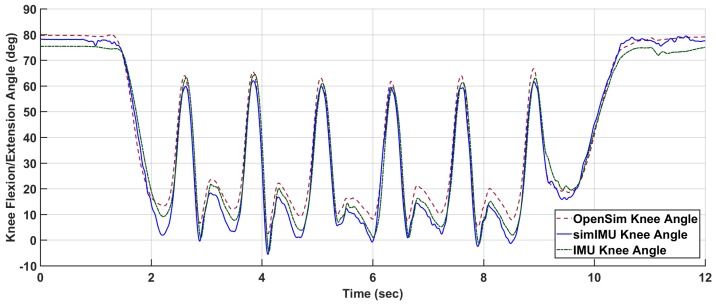
Example absolute knee angle data, shown for subject Y2, TUGT trial 6. Shown are the measured IMU data (IMU, absolute RMSE = 5.35${}^{\circ }$, zero-mean RMSE = 4.58${}^{\circ }$) and simulated IMU data from the marker triads (simIMU, absolute RMSE = 5.84${}^{\circ }$, zero-mean RMSE = 2.94${}^{\circ }$) against the OpenSim knee angle truth.

For each trial, time-series measurements were compared: the ground-truth knee flexion/extension angle according to OpenSim inverse kinematics and the proposed method formulated in frame B. An example of these data can be seen in Figure 2. The IMUs and Vicon system were synchronized in time using an external trigger for subjects 9 and 11–15. For all other subjects, the IMU-estimated knee angle and OpenSim-modeled knee angle were synchronized in time via cross-correlation. For purposes of bounding the beginning and end of the trial for IMU data, the trial was defined to begin when the marker on top of the subject’s head first reached 300 mm/s vertical velocity, indicating that the subject was in the process of standing. Likewise, the trial was defined to end when the subject finally attained 300 mm/s of downward velocity for the same marker, indicating that the subject was in the process of sitting. This parameter was found from empirical tuning to work for all subjects, and included very minimal non-gait motion.

Finally, it is noted that IMU orientation estimate is imperfect, especially in indoor settings. Erroneous orientation estimates of the IMU will induce error into the IMU-estimated knee angle—an error which is not due to the proposed method. In order to control for this error, IMU data was also simulated using the marker triads on the IMUs shown in Figure 1. As the proposed method is agnostic to the alignment of the coordinate systems to the leg segment, the simulated IMU coordinate system can be constructed from the marker triad in any orientation. The relative orientation between the marker triad coordinate system and the Vicon global frame can then be determined. From the simulated IMU orientation and the Vicon sampling rate, the discrete angular velocity was determined. This orientation and angular velocity was then used in the proposed method just as measured IMU angular velocity and estimated orientation would be. An example of the knee angle from this simulated IMU data with the proposed method is also included in Figure 2. 

### 3.4. Measures Collected

It was previously hypothesized in Section 1 hypothesis 2 that IMU location and soft tissue noise may affect the accuracy of the method. This is motivated by the amount of soft tissue artifacts—fat tissue and muscle contraction—of the leg seeming to vary across the lateral-to-anterior surface. Due to the contraction of the quadriceps, we postulate that an IMU on the anterior surface would experience more perturbation relative to the underlying bone structure than an IMU on the lateral surface of the thigh. This relative motion violates the rigid body assumption of the method.

**Figure 3:**
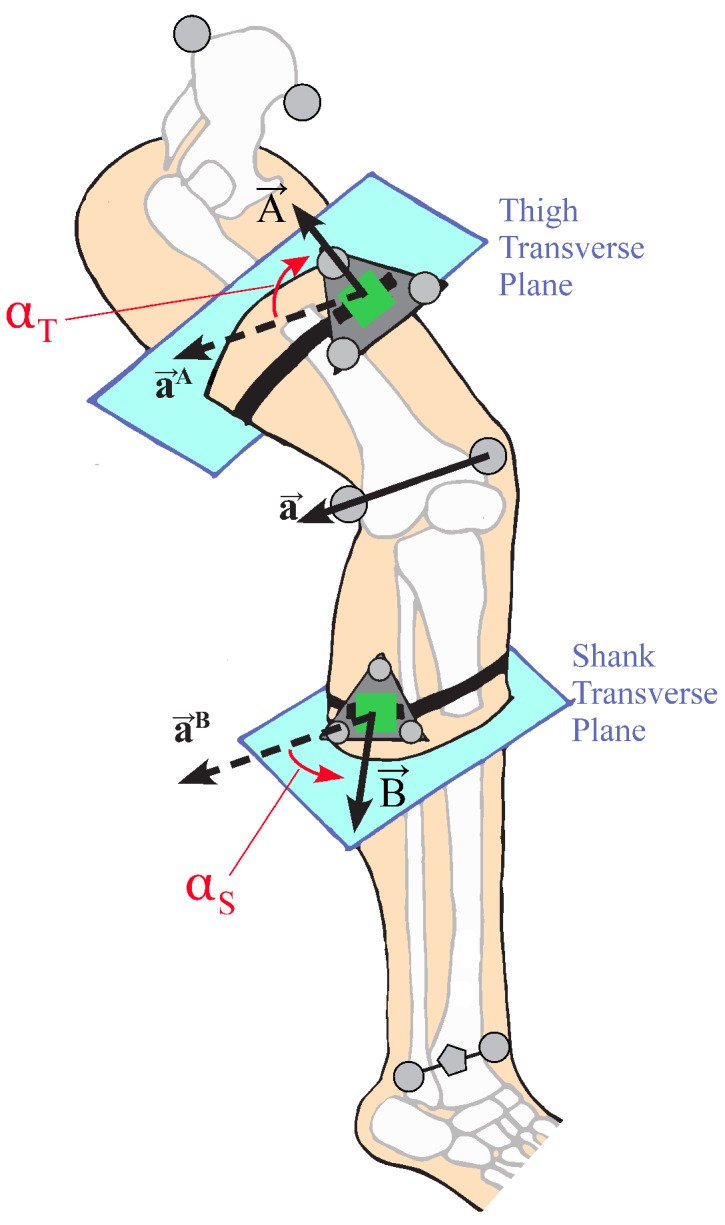
Illustration of the circumferential angle of the IMU (green) on the thigh ($\alpha_{T}$) and shank ($\alpha_{S}$). The knee axis ${\vec{a}}^{}$ and its representation in the transverse planes of the thigh and shank are also shown. The pentagonal marker between the medial/lateral ankle markers represents the virtual marker that was used with the two knee markers to define the coronal plane of the shank segment. $\vec{A}$ and $\vec{B}$ represent the projection of the IMU normal vectors into the transverse planes of the thigh and shank, respectively.

In order to analyze this hypothesis, the circumferential placement of the IMU was defined as the angle between the IMU normal vector and the lateral direction of the leg segment, measured in the transverse plane normal to the body segment (Figure 3). The transverse plane of the body segment is defined as mutually orthogonal to the sagittal and coronal planes. The coronal plane of the thigh is defined as the plane that contains the medial and lateral knee markers and the hip joint center, calculated by relationships to pelvic anthropometry as suggested by Seidel et al. [43]. The coronal plane of the shank contains the two knee markers and the centerpoint of the medial and lateral ankle markers. The sagittal planes of both leg segments are defined as the plane whose normal runs through both knee markers. The circumferential angle α of the IMU is then defined as the angle between the lateral-pointing vector of the body segment in the transverse plane, and the IMU normal vector projected into the transverse plane. The circumferential angle for the thigh IMU would be calculated as:

(5) $$\alpha_{T}=atan2\left(∥{\vec{a}}^{A}\times \vec{A}∥,{\vec{a}}^{A}\cdot \vec{A}\right)$$

and similarly for the shank IMU, where $\vec{A}$ represents the projection of the thigh IMU normal vector into the transverse plane. For example, an IMU placed perfectly on the lateral surface of a body segment would be at $\alpha =0^{\circ }$, whereas an IMU placed perfectly on the anterior surface of the thigh or shank would be at $\alpha =90^{\circ }$. Note that, for the calculation of α in this study, the IMU marker triads were used to define the IMU normal vector, and the knee hinge axis ${\vec{a}}^{}$ was determined from the medial and lateral knee markers.

**Figure 4:**
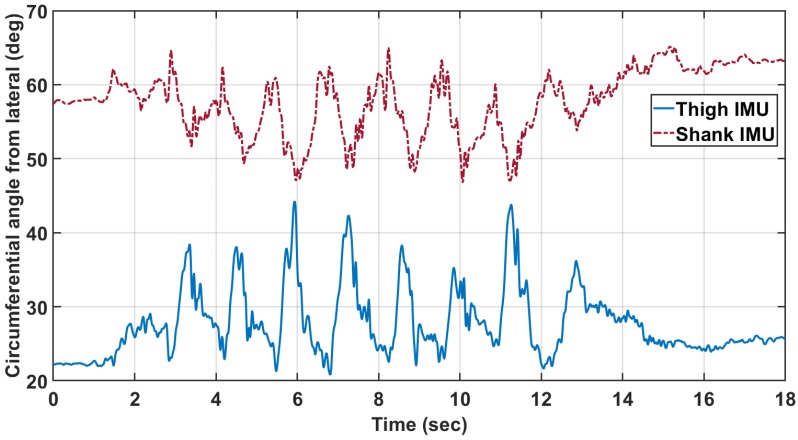
The circumferential angles of the IMU placement on the thigh ($\bar{\alpha_{T}}=57.0^{\circ }$, $\sigma_{\alpha_{T}}=4.2^{\circ }$) and shank ($\bar{\alpha_{S}}=26.7^{\circ }$, $\sigma_{\alpha_{S}}=4.3^{\circ }$) for Subject Y1 TUGT trial 6.

The circumferential angle was computed as a function of time for every trial. It was found that the angle varied significantly over the course of the trial (Figure 4). The general position of the IMUs on the leg segment were quantified as the mean of the circumferential angle over time, and the standard deviation is used as an analog for the amount of motion relative to the rigid body bone structure that the IMU experienced. The mean and standard deviation of the circumferential angle will be denoted as $\bar{\alpha_{T}}$ and $\sigma_{\alpha_{T}}$, respectively, for the thigh. Likewise, the circumferential angle measures for the shank are denoted as $\bar{\alpha_{S}}$ and $\sigma_{\alpha_{S}}$.

It was further hypothesized that subjects with more fatty tissue would induce more perturbations to the IMU, and thus more error. While measures of subject BMI were not collected in this study, the ratio of a subject’s thigh width-to-length was used as a surrogate measure. For each subject, this measure will be denoted as $W/L_{thigh}$.

Finally, it was hypothesized that with poor knee axis estimation comes poor knee angle estimation. Within a trial, the simulated IMU-estimated knee axis was compared with the knee axis as defined by the medial and lateral knee markers. The median of this axis estimation error was also used as a predictor for knee angle error, denoted as $\epsilon_{axis}$.

### 3.5. Statistical Analysis

Data were analyzed for global results via the Bland–Altman method [44,45], a statistical method to evaluate the agreement of two multi-sample measurements. Beyond the overall estimation of error RMSE, Bland–Altman analysis can enable inferences about the linearity of the residuals over the measurement domain. Bland–Altman analysis is usually represented in two plots, the first of the two plots will show a linear regression of the simulated IMU-estimated knee angle vs. the OpenSim-modeled motion capture knee angle. These would be ideally linearly correlated with a unity slope. The second plot will show the difference between measurements on the vertical axis and the average of the two measurements on the horizontal axis. These deviations would ideally have zero mean and a low variance.

All of the knee angles according to OpenSim and the proposed method with simulated IMU data were concatenated for error calculation and Bland–Altman analysis to create a composite assessment of all subjects. Measurement error over the entire study will be reported in terms of overall $r^{2}$ linearity coefficient, static bias of the measurement error with probability of that bias being non-zero, and 95% confidence interval of the error.

A post hoc linear mixed effects model was constructed to investigate factors that contribute to RMSE, here we specifically consider the zero-mean RMSE of the simulated IMU method as this model would highlight the effects of the factors on the PCA methodology. RMSE per trial was modeled as a linear function of the aforementioned fixed-effect factors: circumferential angle mean and standard deviation for the thigh and shank, thigh-width-to-length ratio, and estimated axis error, along with relevant interaction effects. As the trials within a subject are not independent, subject is included as a random effect, modeled as a random intercept. Factors which did not have a nearly-significant main effect or interaction effect were then removed from the model. This reduced model is presented in the results section. When fitting the model, fixed factors were removed from the model if the estimated slopes of the interaction and main effect were not significant at α=0.1; however, for interpretation, we consider significance as α=0.05. These factors were removed to prevent overfitting of the model.

## 4. Results and Discussion

### 4.1. Overall Performance

**Figure 5:**
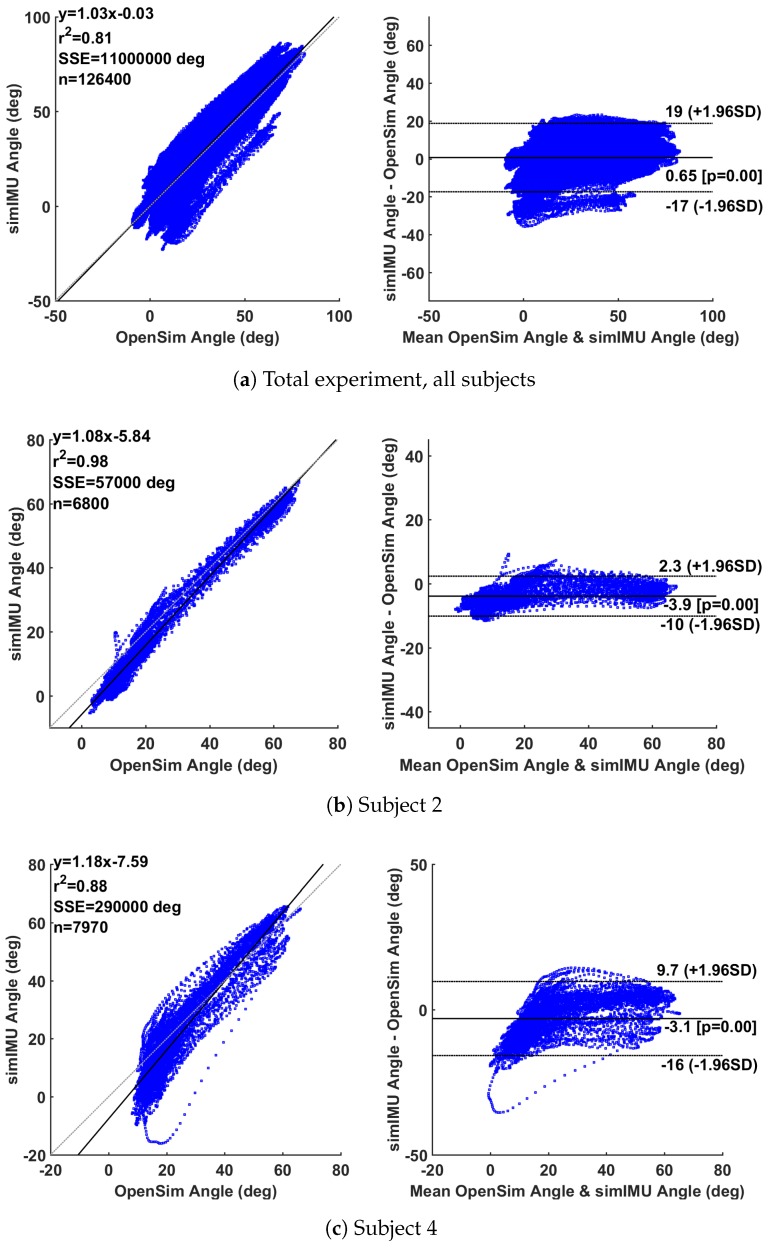
Selected Bland–Altman analyses; linear model given by y(x) with associated squared correlation coefficient $r^{2}$, including sum of squared error (SSE) over *n* data points. Estimated data is the knee angle according to the proposed method on the simulated IMU data.

Analysis of the simulated IMU data through the proposed method formulated in frame B showed an absolute RMSE of 9.24${}^{\circ }$ and a zero-mean RMSE of 3.49${}^{\circ }$. Bland–Altman analysis of the total experiment (using the absolute simulated IMU data) in Figure 5a shows a linearity of $r^{2}$ = 0.81 to the best-fit model y=1.03x−0.03, a 95% confidence interval of the error on [+19,−17]${}^{\circ }$, and an average static bias offset of 0.65${}^{\circ }$ (p<0.001). There was high variability between subjects, for example subject Y2 in Figure 5b with $r^{2}$ = 0.98, [+2.3${}^{\circ }$,−10${}^{\circ }$] confidence interval, −3.9${}^{\circ }$ average offset (p<0.001), and subject Y4 in Figure 5c with $r^{2}$ = 0.88, [+9.7${}^{\circ }$,−16${}^{\circ }$] confidence interval, and −3.1${}^{\circ }$ average offset (p<0.001).

For comparison, the Seel et al. [30] axis estimation method was also implemented with the angle calculation approach described in Section 2. The RMSE of the proposed method by subject is shown in Table 1. The difference in results between the proposed method on the simulated IMU data and the measured IMU data with the manufacturer’s onboard orientation estimator shows that there was significant error in the IMU’s orientation estimation in the lab environment. Likewise, the difference between the absolute RMSE and the zero-mean RMSE of the proposed method on the simulated IMU data suggests some disagreement between the knee angle datums of the OpenSim-estimated knee angle and the IMU-based knee angle. These analyses suggest a good level of agreement between the proposed method and the OpenSim-modeled optical motion capture measurement method. As compared to a traditional motion capture system, this method can be implemented in the field, rather than being constrained to a laboratory environment. Likewise, as compared to traditional IMU-based joint angle measurement approaches, this method assumes no alignment of the IMU to a limb segment. As a result, this method would be easier and faster to implement for both researchers and non-experts alike. The accuracy of the proposed system is similar to that of previous literature [11,16], without the need to align the system to body segments or perform a prior calibration. There was some variation in the quality of this knee angle estimation between subjects, as illustrated in Figure 5 and Table 1.

Bland–Altman analysis on some individual subjects may also suggest a nonlinearity at the low end of the measurement domain, corresponding to near-full extension of the knee. It seemed that the measurement system captured the knee angle well throughout much of gait, but at full extension the “true” measurement and the IMU measurement do not agree well (see Figure 5b,c). Generally, error in knee angle estimation can be attributed to the violation of the method’s basic assumption: that the knee hinge axis is time-invariant and can be represented statically in both IMU frames. A few different factors may contribute the violation of this assumption, namely, (1) that the knee flexion/extension axis is not a perfectly time-invariant hinge; and (2) perturbations to the IMUs can cause artificial movement of the IMU frame, thus causing the static estimate of the knee axis to deviate from the true axis. These perturbations of the IMUs could be due, for example, to soft tissue artifacts on the leg. Near full-extension of the knee, the quadriceps contract and cause the anterior surface of the thigh to expand. An IMU on the surface of the thigh would likewise bulge out. Furthermore, near full-extension of the knee, the foot contacts the ground in gait, possibly causing vibrations in the tissue of the leg, which would transfer to the IMU. The following section investigates the effect of IMU perturbation, axis estimation accuracy, and subject anthropometry on the knee angle estimation error.

The Favre et al. [17] IMU measurement system estimated knee flexion/extension angle to an accuracy of 1.5${}^{\circ }$ mean RMSE after a calibration procedure including a static pose and prescribed hip abduction motion. The Seel et al. [30] auto-calibrating IMU measurement system reported an error of 3.3${}^{\circ }$ RMSE for a single subject performing six gait trials. The present findings suggest that evaluation of wearable IMU methods on larger subject populations is important for method characterization. This work extends the existing literature by evaluating an auto-calibrating knee IMU-based measurement system on a larger subject set, studying the operational predictors to error in an auto-calibrating measurement system, and the proposal and implementation of a computationally-inexpensive algorithm that is robust to sign-ambiguity problems common to most axis estimation approaches. The variability in error across subjects underscores the importance of testing wearable IMU methods with larger subject populations.

### 4.2. Explanatory Model

**Figure 6:**
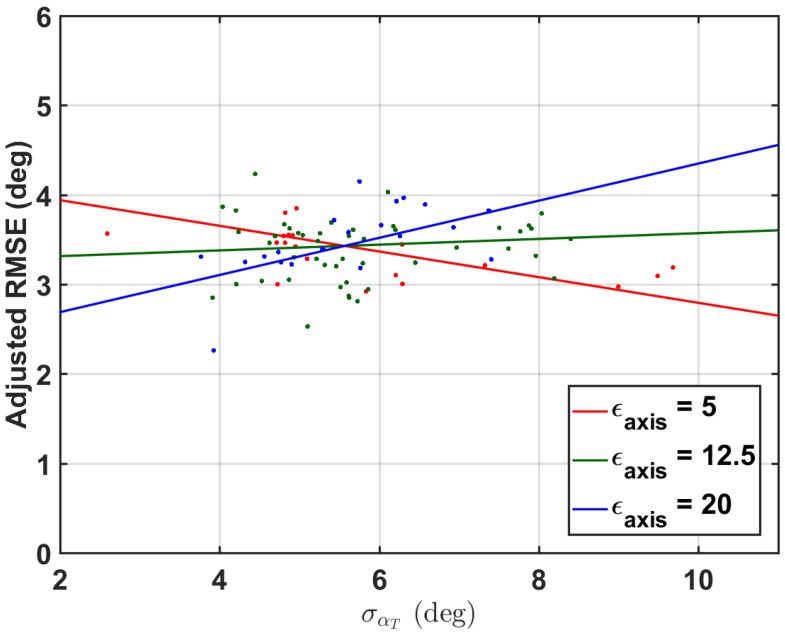
The linear model, plotted as a function of $\sigma_{\alpha_{T}}$ and $\epsilon_{axis}$. The points selected have been adjusted by the estimated subject intercept (random effect) so that multiple subjects can be presented with the same linear regression lines (plotted using only the fixed effects and Constant). The points selected for each line were those that had $\epsilon_{axis}\pm 2.5^{\circ }$ about the selected value.

Measures collected to test the hypothesis 2 of Section 1 are described in Section 3.4. These measures were used as fixed-effects in a linear mixed effects model to predict zero-mean RMSE of the simulated IMU data with the proposed method within a trial. Appropriate two-way interaction terms were included. The subject was also included as a random effect. The results of the reduced linear model are shown in Table 2. The overall model adjusted $R^{2}=0.85$ suggests a good linear fit, to a total significance of p=0.004 (F(3,139)=4.59). The model is shown in Figure 6.

While the main effects of the model suggest a significance of $\sigma_{\alpha_{T}}$ and $\epsilon_{axis}$ to the linear model predicting zero-mean RMSE of the proposed method on the simulated IMU data, the interaction effect dominates the main effect. The interaction of $\sigma_{\alpha_{T}}$ to $\epsilon_{axis}$ lends that there is a larger increase in RMSE with more $\sigma_{\alpha_{T}}$ when the axis is poorly estimated, i.e., good axis estimation makes the method more robust to increased $\sigma_{\alpha_{T}}$. However, the effect size is clearly small. Researchers can probably gain more in the way of method accuracy through improving IMU orientation estimation rather than specific sensor placement. These results corroborate those of Graurock et al. [46] who found little operational difference between sensor placements on the thigh. However, they do still recommend placing the sensors on the lateral side of the thigh.

### 4.3. Future Work and Limitations

Future work will seek to implement a filter-based estimator for the knee hinge axis and angle, allowing the axis to be estimated over time in a statistically-robust manner. Furthermore, as the estimated knee angle seems to deviate from truth near the extremes of the knee flexion/extension domain, the measurement error may be able to be reduced by modeling the error as a possibly nonlinear function of measured knee angle. Finally, the knee hinge axis is not perfectly fixed; relaxing this assumption to estimate the time-variant knee hinge axis may also reduce measurement error.

The predictors of the linear model were not varied as factors in the observational experiment; instead, they were measured post hoc to build the explanatory model, leading to a limited region of the parameter space for which the model is appropriate. While this analysis considers a reasonable domain, follow-on factorial studies could explore error dependence over a wider parameter space. A future experiment could experimentally vary these factors and take proper body fat measures to more robustly understand the factors affecting the error of the proposed method. Furthermore, in this model, axis error was quantified as the difference between method-estimated knee hinge axis and the knee axis as defined by the medial and lateral knee markers. There is the potential for small shifts in marker placement, which would affect these results.

It should be noted that the proposed method requires orientation estimation of two IMUs to a common global frame. In practice, there are always some errors associated with orientation estimation. Some errors in this process could be due to estimating IMU orientation relative to slightly different world frames. Methods have been proposed to remedy this problem [27,41]. The Seel et al. [30] method does not require orientation estimation to estimate the knee hinge axis or flexion/extension angle, which could prove advantageous in high-orientation-estimation error situations. While this work relied on the onboard orientation estimation algorithm from the manufacturer, this method could be employed with any orientation estimation technique. Further estimation methods have been proposed [47] to aid in the general problem of magnetometer fusion in the presence of variable magnetic fields (i.e., many indoor scenarios). The proposed PCA method shows promise with accurate IMU orientation estimation. Finally, the presented angle decomposition method is only appropriate for calculation of knee flexion/extension angle due to selection of an arbitrary *x*-axis. Interpretation of the other rotations as meaningful knee angles would require estimation of the other rotation axes of the knee.

Comparison of this method to an OpenSim truth also required understanding of the biomechanical datum for knee flexion/extension angle present in both the OpenSim knee angle and the IMU-estimated knee angle. These datums may be different due to small shifts in marker placement on the subject or IMU placement such that the IMUs do not align with the underlying biomechanical model of the leg segment. This limitation motivated the reporting of RMSE as both the absolute RMSE and zero-mean RMSE. The zero-mean RMSE is a better comparison of the difference between true and estimated knee angle waveforms, while the absolute RMSE would include this difference and also static offset errors due to different datums between the OpenSim knee angle and estimated knee angle.

## 5. Conclusions

A method has been developed for the estimation of the knee flexion/extension angle using inertial sensors. This method allows for estimation of the angle without cumbersome alignments of the IMUs to the leg segments, as the system calibrates itself from any general hinge motion of the knee. The knee’s major rotation axis is estimated through the use of PCA, a computationally-efficient and simple algorithm that can be implemented online that is robust to the positive/negative sign ambiguity of the estimated knee axis. When pooling all subjects, the method performed with absolute RMSE of 9.24${}^{\circ }$ and a zero-mean RMSE of 3.49${}^{\circ }$ as compared to an optical motion capture gold standard, although subjects ranged between 2.32${}^{\circ }$ and 4.86${}^{\circ }$ zero-mean RMSE. Unlike previous literature, the method is evaluated over a larger population (15 subjects), and variability in RMSE across subjects was found.

In addition, an explanatory model of the error has been presented. The model suggests that method error is a function of thigh IMU circumferential motion and quality of knee flexion/extension hinge axis estimation—highlighting the importance of axis estimation to calculation of knee angle. However, the effect size for these parameters is small in comparison to potential gains from improved IMU orientation estimations. Furthermore, the results underscore the importance of understanding and setting appropriate datums when comparing two differently-measured data series.

## Figures and Tables

**Table 1:** RMSE in degrees by subject for selected analyses: zero-mean RMSE of the simulated IMU knee angle with the proposed method, zero-mean RMSE of the actual IMU data with the proposed method, zero-mean RMSE of the Seel et al. method [30] on the simulated IMU data, and absolute RMSE of the simulated IMU data with the proposed method.

Subject	RMSE${}_{0}\;$ (simIMU)	RMSE${}_{0}\;$ (IMU)	RMSE${}_{0}\;$ (simSeel)	Absolute RMSE (simIMU)
1	2.32	4.83	2.96	15.11
2	3.00	4.53	3.69	5.03
3	2.89	6.49	7.23	6.26
4	4.86	5.42	5.20	7.19
5	4.73	8.54	6.56	8.33
6	2.90	4.10	6.01	3.63
7	2.50	6.14	6.45	14.36
8	2.77	12.93	7.70	7.08
9	3.56	6.51	9.87	8.21
10	3.38	13.44	4.50	16.01
11	3.79	11.27	6.67	4.11
12	3.89	16.97	7.52	10.84
13	3.46	13.75	7.00	4.53
14	3.58	6.01	4.49	6.34
15	4.25	14.93	7.95	6.28
Total	3.49	9.69	6.42	9.24

**Table 2:** All terms in the reduced linear model. Significant parameters included thigh circumferential angle standard deviation ($\sigma_{\alpha_{T}}$), median axis estimation error ($\epsilon_{axis}$), and the interaction between the two. Estimate is the estimated coefficient for the predictor variable (intercept or slope coefficient), SE is the standard error of the estimate, *t* is the associated *t*-statistic of the coefficient against the null hypothesis of a zero value, and *p* is the *p*-value associated with the *t*-statistic. Significance was set as p<0.05.

Term	Estimate	SE	*t*	*p*
Constant	4.878	0.544	8.961	<0.001
$\sigma_{\alpha_{T}}$	−0.260	0.083	−3.136	0.002
$\epsilon_{axis}$	−0.130	0.041	−3.143	0.002
$\sigma_{\alpha_{T}}\times \epsilon_{axis}$	0.023	0.007	3.559	<0.001

## References

[1] Yang Y., Leung H., Yue L., Deng L. (2010). Evaluating Human Motion Complexity Based on Un-Correlation and Non-Smoothness.

[2] Ceseracciu E., Sawacha Z., Cobelli C. (2014). Comparison of markerless and marker-based motion capture technologies through simultaneous data collection during gait: Proof of concept. PLoS ONE.

[3] Moeslund T.B., Granum E. (2001). A Survey of Computer Vision-Based Human Motion Capture. Comput. Vis. Image Underst..

[4] Delp S.L., Anderson F.C., Arnold A.S., Loan P., Habib A., John C.T., Guendelman E., Thelen D.G. (2007). OpenSim: Open source to create and analyze dynamic simulations of movement. IEEE Trans. Biomed. Eng..

[5] Sabatini A.M. (2006). Quaternion-based extended Kalman filter for determining orientation by inertial and magnetic sensing. IEEE Trans. Biomed. Eng..

[6] King A.D. (1998). Inertial navigation—Forty years of evolution. GEC Rev..

[7] Harada T., Uchino H., Mori T., Sato T. Portable orientation estimation device based on accelerometers, magnetometers and gyroscope sensors for sensor network. Proceedings of the IEEE International Conference on Multisensor Fusion and Integration for Intelligent Systems.

[8] Roetenberg D., Luinge H.J., Baten C.T.M., Veltink P.H. (2005). Compensation of magnetic disturbances improves inertial and magnetic sensing of human body segment orientation. IEEE Trans. Neural Syst. Rehabil. Eng..

[9] Elwell J. Inertial navigation for the urban warrior. Proceedings of the SPIE 3709, Digitization of the Battlespace IV.

[10] Luinge H.J., Veltink P.H., Baten C.T.M. (1999). Estimating orientation with gyroscopes and accelerometers. Technol. Health Care.

[11] Mayagoitia R.E., Nene A.V., Veltink P.H. (2002). Accelerometer and rate gyroscope measurement of kinematics: An inexpensive alternative to optical motion analysis systems. J. Biomech..

[12] Boone D.C., Azen S.P. (1979). Normal range of motion of joints in male subjects. J. Bone Jt. Surg. Am. Vol..

[13] Zhang L.Q., Xu D., Wang G., Hendrix R.W. (2001). Muscle strength in knee varus and valgus. Med. Sci. Sports Exerc..

[14] Lafortune M.A., Cavanagh P.R., Sommer H.J., Kalenak A. (1992). Three-dimensional kinematics of the human knee during walking. J. Biomech. Eng..

[15] Fitzgerald G.K., Piva S.R., Irrgang J.J. (2004). Reports of joint instability in knee osteoarthritis: Its prevalence and relationship to physical function. Arthritis Care Res..

[16] Favre J., Luthi F., Jolles B.M., Siegrist O., Najafi B., Aminian K., Ne K., Favre E.J., Najafi A.B., Aminian A.K. (2006). A new ambulatory system for comparative evaluation of the three-dimensional knee kinematics, applied to anterior cruciate ligament injuries. Knee Surg. Sports Traumatol. Arthrosc..

[17] Favre J., Jolles B.M., Aissaoui R., Aminian K. (2008). Ambulatory measurement of 3D knee joint angle. J. Biomech..

[18] Barré A., Thiran J.P., Jolles B.M., Theumann N., Aminian K. (2013). Soft tissue artifact assessment during treadmill walking in subjects with total knee arthroplasty. IEEE Trans. Biomed. Eng..

[19] Grood E.S., Suntay W.J. (1983). A Joint Coordinate System for the Clinical Description of Three-Dimensional Motions: Application to the Knee. J. Biomech. Eng..

[20] Wu G., Siegler S., Allard P., Kirtley C., Leardini A., Rosenbaum D., Whittle M., D’Lima D.D., Cristofolini L., Witte H. (2002). ISB recommendation on definitions of joint coordinate system of various joints for the reporting of human joint motion—Part I: Ankle, hip, and spine. J. Biomech..

[21] Wu G., Van Der Helm F.C., Veeger H.E., Makhsous M., Van Roy P., Anglin C., Nagels J., Karduna A.R., McQuade K., Wang X. (2005). ISB recommendation on definitions of joint coordinate systems of various joints for the reporting of human joint motion—Part II: Shoulder, elbow, wrist and hand. J. Biomech..

[22] Favre J., Jolles B., Siegrist O., Animian K. (2006). Quaternion-based fusion of gyroscopes and accelerometers to improve 3D angle measurement. Electron. Lett..

[23] Zhu R., Zhou Z. (2004). A real-time articulated human motion tracking using tri-axis inertial/magnetic sensors package. IEEE Trans. Neural Syst. Rehabil. Eng..

[24] Luinge H.J., Veltink P.H., Baten C.T.M. (2007). Ambulatory measurement of arm orientation. J. Biomech..

[25] Cutti A.G., Ferrari A., Garofalo P., Raggi M., Cappello A., Ferrari A. (2010). ‘Outwalk’: A protocol for clinical gait analysis based on inertial and magnetic sensors. Med. Biol. Eng. Comput..

[26] Favre J., Aissaoui R., Jolles B.M., de Guise J.A., Aminian K. (2009). Functional calibration procedure for 3D knee joint angle description using inertial sensors. J. Biomech..

[27] Vitali R.V., Cain S.M., McGinnis R.S., Zaferiou A.M., Ojeda L.V., Davidson S.P., Perkins N.C. (2017). Method for estimating three-dimensional knee rotations using two inertial measurement units: Validation with a coordinate measurement machine. Sensors.

[28] Cooper G., Sheret I., McMillian L., Siliverdis K., Sha N., Hodgins D., Kenney L., Howard D. (2009). Inertial sensor-based knee flexion/extension angle estimation. J. Biomech..

[29] Seel T., Schauer T. Joint Axis and Position Estimation from Inertial Measurement Data by Exploiting Kinematic Constraints. Proceedings of the 2012 IEEE International Conference on Control Applications (CCA).

[30] Seel T., Raisch J., Schauer T. (2014). IMU-based joint angle measurement for gait analysis. Sensors.

[31] Muller P., Begin M.A., Schauer T., Seel T. (2016). Alignment-Free, Self-Calibrating Elbow Angles Measurement using Inertial Sensors. IEEE J. Biomed. Health Inf..

[32] Hotelling H. (1933). Analysis of a complex of statistical variables into principal components. J. Educ. Psychol..

[33] Daffertshofer A., Lamoth C.J., Meijer O.G., Beek P.J. (2004). PCA in studying coordination and variability: A tutorial. Clin. Biomech..

[34] Landry S.C., McKean K.A., Hubley-Kozey C.L., Stanish W.D., Deluzio K.J. (2007). Knee biomechanics of moderate OA patients measured during gait at a self-selected and fast walking speed. J. Biomech..

[35] Dillmann U., Holzhoffer C., Johann Y., Bechtel S., Gräber S., Massing C., Spiegel J., Behnke S., Bürmann J., Louis A.K. (2014). Principal Component Analysis of gait in Parkinson’s disease: Relevance of gait velocity. Gait Posture.

[36] McGrath T., Stirling L. Calibration-free, online estimation of the knee flexion/extension axis using inertial measurement units. Proceedings of the IEEE/RSJ International Conference on Intelligent Robots and Systems.

[37] Kear B.M., Guck T.P., McGaha A.L. (2016). Timed Up and Go (TUG) Test. J. Prim. Care Community Health.

[38] Sabatini A.M. (2011). Kalman-filter-based orientation determination using inertial/magnetic sensors: Observability analysis and performance evaluation. Sensors.

[39] Madgwick S.O.H., Harrison A.J.L., Vaidyanathan R. Estimation of IMU and MARG orientation using a gradient descent algorithm. Proceedings of the IEEE International Conference on Rehabilitation Robotics.

[40] Yun X., Bachmann E. (2006). Design, Implementation, and Experimental Results of a Quaternion-Based Kalman Filter for Human Body Motion Tracking. IEEE Trans. Robot..

[41] Laidig D., Schauer T., Seel T. Exploiting kinematic constraints to compensate magnetic disturbances when calculating joint angles of approximate hinge joints from orientation estimates of inertial sensors. Proceedings of the IEEE International Conference on Rehabilitation Robotics.

[42] Delp S.L., Loan J.P., Hoy M.G., Zajac F.E., Topp E.L., Rosen J.M. (1990). An Interactive Graphics-Based Model of the Lower Extremity to Study Orthopaedic Surgical Procedures. IEEE Trans. Biomed. Eng..

[43] Seidel G.K., Marchinda D.M., Dijkers M., Soutas-Little R.W. (1995). Hip joint center location from palpable bony landmarks—A cadaver study. J. Biomech..

[44] Myles P.S., Cui J.I. (2007). Using the Bland–Altman method to measure agreement with repeated measures. Br. J. Anaesth..

[45] Bland J., Altman D. (1986). Statistical Methods for Assessing Agreement Between Two Methods of Clinical Measurement. Lancet.

[46] Graurock D., Schauer T., Seel T. User-Adaptive Inertial Sensor Network for Feedback-Controlled Gait Support Systems. Proceedings of the International Functional Electrical Stimulation Society.

[47] Seel T., Ruppin S. (2017). Eliminating the Effect of Magnetic Disturbances on the Inclination Estimates of Inertial Sensors. IFAC PapersOnLine.

