# Correction: McGrath, T., et al. An Auto-Calibrating Knee Flexion-Extension Axis Estimator using Principal Component Analysis with Inertial Sensors. *Sensors* 2018, 18(6), 1882

**DOI:** 10.3390/s19071504

**Published:** 2019-03-28

**Authors:** Timothy McGrath, Richard Fineman, Leia Stirling

**Affiliations:** 1 Department of Aeronautics and Astronautics, Massachusetts Institute of Technology, 77 Massachusetts Avenue, Cambridge, MA 02139, USA; leia@mit.edu; 2 Harvard-MIT Division of Health Sciences & Technology, Massachusetts Institute of Technology, 77 Massachusetts Avenue, Cambridge, MA 02139, USA; rfineman@mit.edu

The authors wish to make the following revisions to this paper [1].

There are two corrections presented in the revised article.

1. Simple edit: An inconsistent notation in Equation (3) was found in the text.
2. There was an error in the analysis code that used a slightly non-orthogonal coordinate system for the joint segment coordinate systems S (related to Equation (2)). This correction in the analysis code created very small numerical differences in the result which do not affect the overall purpose, conclusions, or discussions of the paper. However, we would like to make these updates for correctness of the presented results.

## 1. Updated Equation (3)

The previous notation of Equation (3) did not use the most common convention. This has been updated to read:

$$D_{S_{1}}^{S_{2}}={\left(D_{S_{2}}^{B}\right)}^{-1}D_{G}^{B}{\left(D_{G}^{A}\right)}^{-1}D_{S_{1}}^{A}$$

## 2. Updated Equation (2)

The previous notation of Equation (2) could potentially produce a non-orthogonal coordinate system, depending on the input data. This has been modified. Replace previous Equation (2):

$$D_{S_{1}}^{A}=\left[\begin{array}{ccc} {\vec{a}}_{y}^{A^{2}}+{\vec{a}}_{z}^{A^{2}} & 0 & {\vec{a}}_{x}^{A}\\ -{\vec{a}}_{x}^{A}{\vec{a}}_{y}^{A} & {\vec{a}}_{z}^{A} & {\vec{a}}_{y}^{A}\\ -{\vec{a}}_{x}^{A}{\vec{a}}_{z}^{A} & -{\vec{a}}_{y}^{A} & {\vec{a}}_{z}^{A} \end{array}\right]\;\forall {\vec{a}}^{A}\neq \left[\begin{array}{ccc} 1 & 0 & 0 \end{array}\right]$$

with updated equation:

$$D_{S_{1}}^{A}=\left[a\;b\;c\right]\;\mathrm{where}\;\mathrm{column}\;\mathrm{vectors}\;c={\vec{a}}^{A},\;b=\frac{{\vec{a}}^{A}\times {\left[1\;0\;0\right]}^{T}}{{∥{\vec{a}}^{A}\times {\left[1\;0\;0\right]}^{T}∥}_{2}},\;a=\left(b\times c\right)$$

## 3. Updating Reported RMSE in Abstract

With new numerical results, root-mean-square error reported in the abstract has been updated from 9.24/3.49 deg absolute/relative to 9.72/3.49 deg.

## 4. Changes to Figure 2

Figure 2 has been updated to represent the new results.

Replace: 
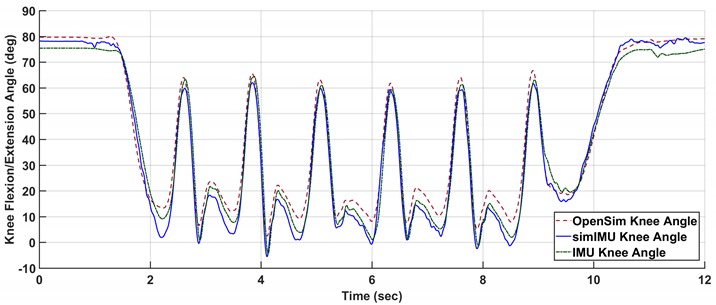


With: 
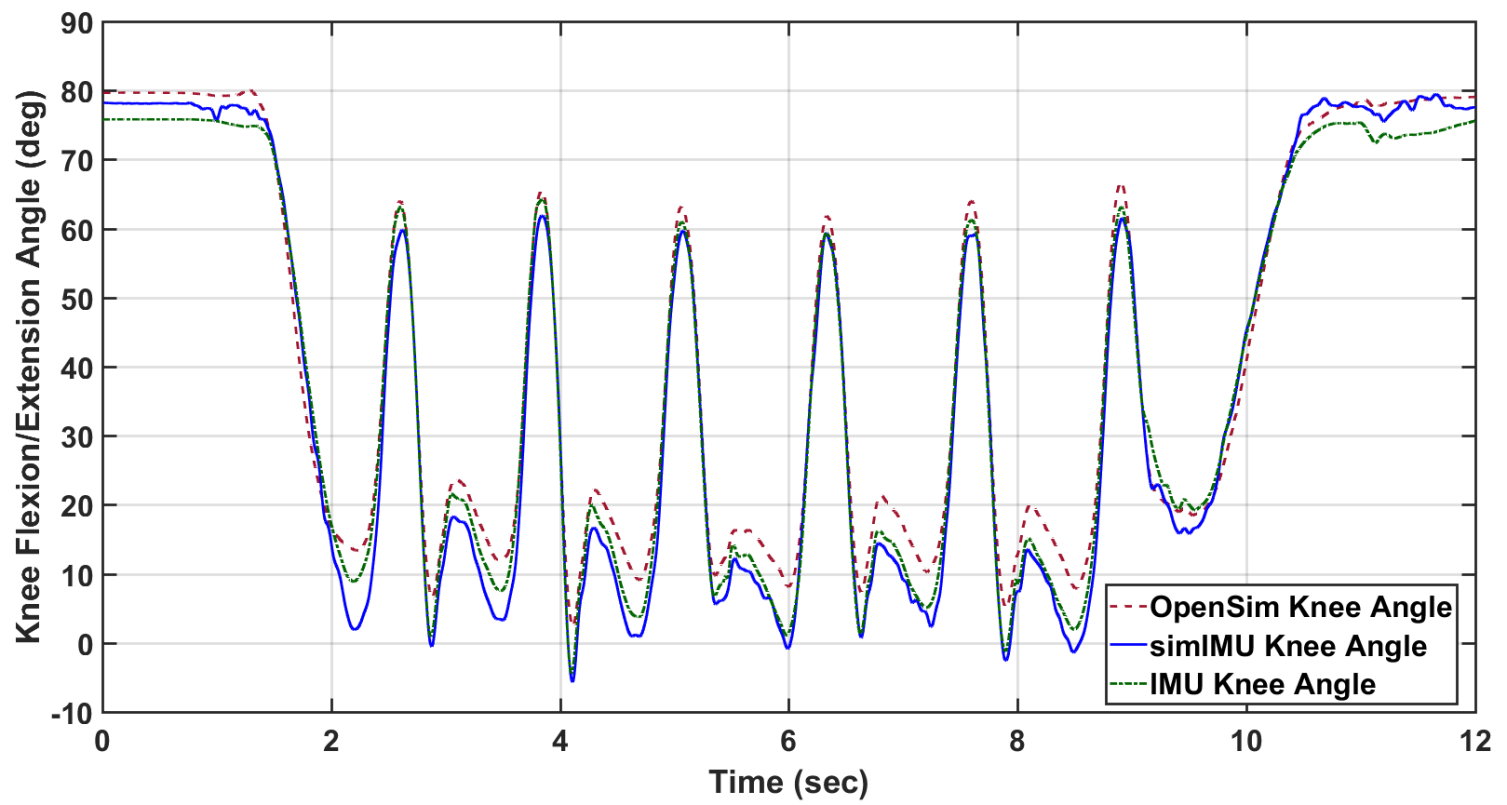


## 5. Changes to Figure 4

Figure 4 has been updated to represent the new results.

Replace: 
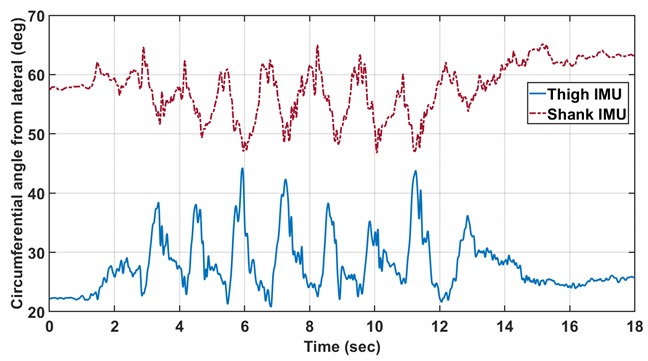


With: 
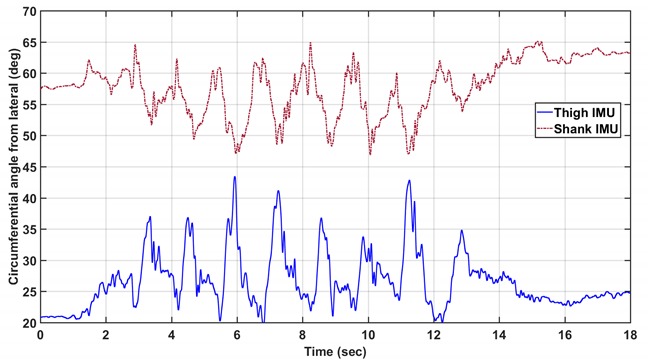


## 6. Updating Results in Section 4.1

Results of statistical analysis in Section 4.1 has been updated to reflect new results.

## 7. Updating RMSE in Table 1

Reported RMSE in Table 1 has been updated.

## 8. Changes in Figure 5

Bland-Altman analysis results reported in Figure 5 have been updated to reflect new data.

Replace the first set of 3 plots with the second set:

## 9. Updating Linear Model Results in Table 2

Table 2 has been updated to reflect the new linear model results. It should now read as:

## 10. Updating Reported Results in Section 4.2

Paragraph 1 in Section 4.2 has been updated to report the new numerical results from the revised Table 2.

## 11. Changes in Figure 6

Figure 6 has been updated to reflect the new explanatory linear model from the revised Table 2.

Replace:

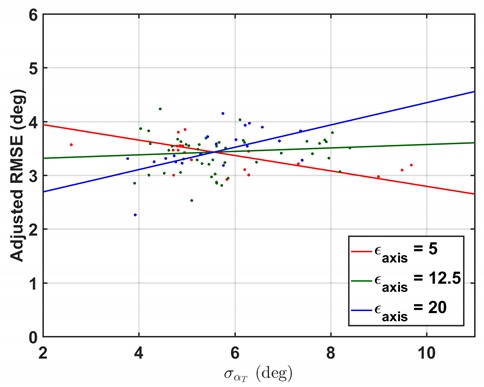



The linear model, plotted as a function of $\sigma_{\alpha_{T}}$ and $\epsilon_{axis}$. The points selected have been adjusted by the estimated subject intercept (random effect) so that multiple subjects can be presented with the same linear regression lines (plotted using only the fixed effects and Constant). The points selected for each line were those that had $\epsilon_{axis}$ ± 2.5° about the selected value.

With:

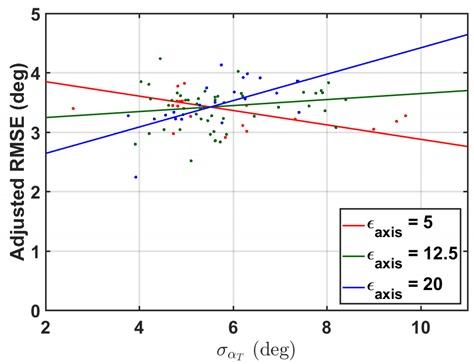



The linear model, plotted as a function of $\sigma_{\alpha_{T}}$ and $\epsilon_{axis}$. The points selected have been adjusted by the estimated subject intercept (random effect) so that multiple subjects can be presented with the same linear regression lines (plotted using only the fixed effects and Constant). The points selected for each line were those that had $\epsilon_{axis}$ ± 2.5° about the selected value.

## 12. Updating RMSE Results in Conclusion

Updated RMSE results in conclusion to reflect new RMSE (as shown in abstract changes section).

## Figures and Tables

**Figure 5:**
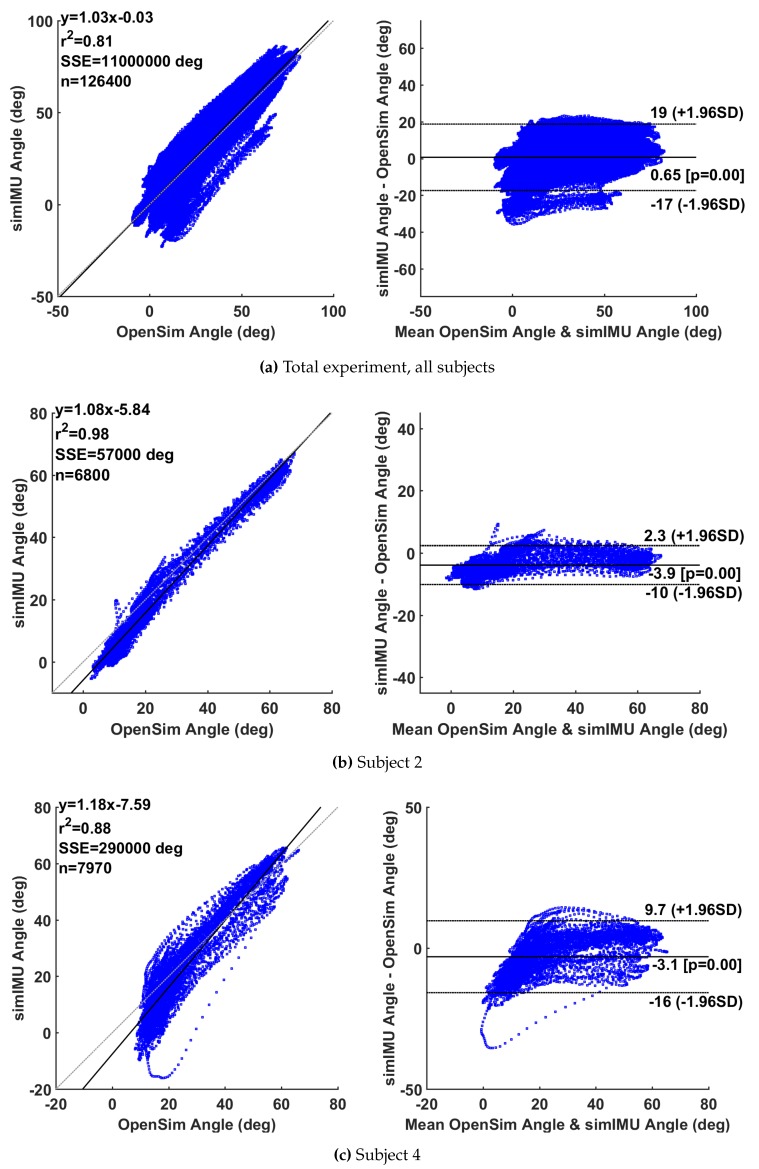
Selected Bland–Altman analyses; linear model given by y(x) with associated squared correlation coefficient $r^{2}$, including sum of squared error (SSE) over *n* data points. Estimated data is the knee angle according to the proposed method on the simulated IMU data.

**Figure 5:**
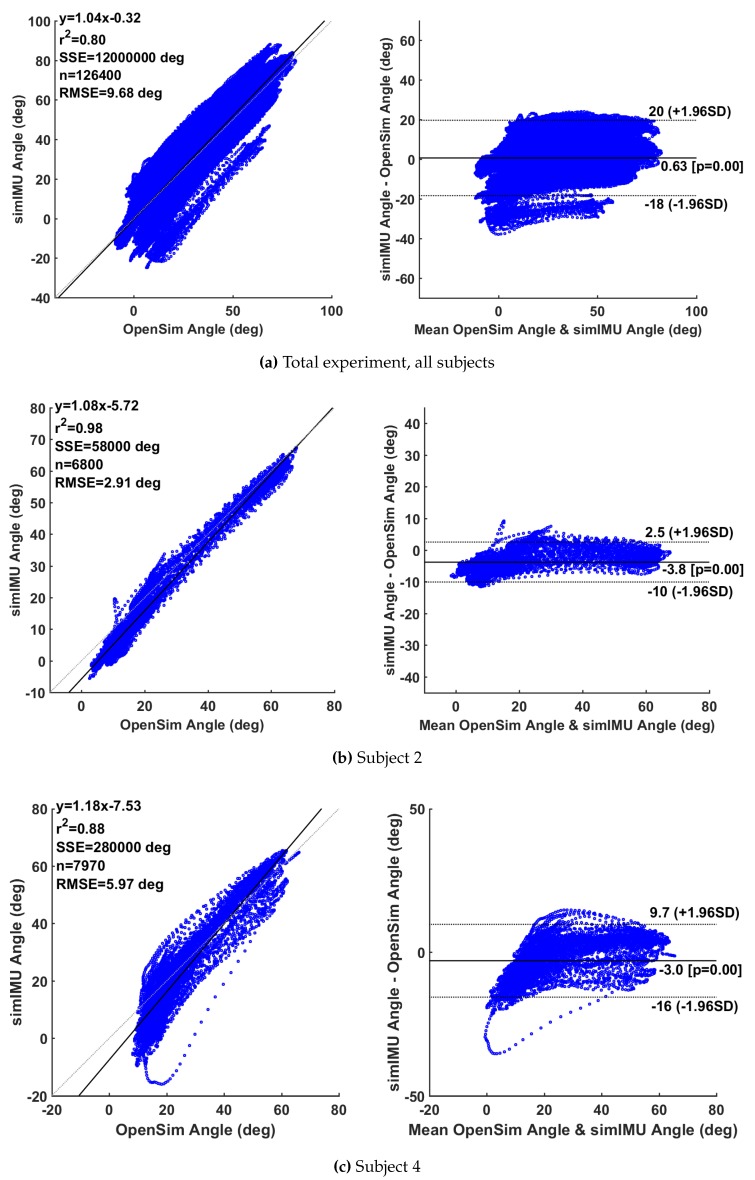
Selected Bland–Altman analyses; linear model given by y(x) with associated squared correlation coefficient $r^{2}$, including sum of squared error (SSE) over *n* data points. Estimated data is the knee angle according to the proposed method on the simulated IMU data.

**Table 2:** All terms in the reduced linear model. Significant parameters included thigh circumferential angle standard deviation ($\sigma_{\alpha_{T}}$), median axis estimation error ($\epsilon_{axis}$), and the interaction between the two. Estimate is the estimated coefficient for the predictor variable (intercept or slope coefficient), SE is the standard error of the estimate, *t* is the associated *t*-statistic of the coefficient against the null hypothesis of a zero value, *p* is the *p*-value associated with the t-statistic. Significance was set as *p* < 0.05.

Term	Estimate	SE	t	p
Constant	4.723	0.546	8.651	<0.001
$\sigma_{\alpha_{T}}$	−0.235	0.083	−2.830	0.005
$\epsilon_{axis}$	−0.126	0.042	−3.040	0.002
$\sigma_{\alpha_{T}}\times \epsilon_{axis}$	0.023	0.007	3.472	<0.001
